# The Health Impact of Intensive and Nonintensive Grandchild Care in Europe: New Evidence From SHARE

**DOI:** 10.1093/geronb/gbv055

**Published:** 2015-08-26

**Authors:** Giorgio Di Gessa, Karen Glaser, Anthea Tinker

**Affiliations:** Institute of Gerontology, Department of Social Science, Health and Medicine, School of Social Science and Public Policy, King’s College London.

**Keywords:** Depressive symptoms, Disability, Europe, Grandparenting, Health, Longitudinal, Self-rated health, SHARE

## Abstract

**Objectives:**

Grandparents are an important source of childcare. However, caring for grandchildren may affect grandparents’ health in both positive and negative ways. Our study examines the association between grandparental childcare and grandparents’ health at 2- and 4-year follow-up.

**Method:**

Our study is based on grandparents aged 50 and older from Waves 1–4 of the Survey of Health, Ageing and Retirement in Europe (SHARE). Using multivariate analyses, we investigated associations between intensive and nonintensive grandparental childcare at Wave 2 and subsequent health (self-rated health, depressive symptoms, and disability) controlling for covariates and health at baseline. Associations between changes over time in grandparental childcare and health at follow-up were also explored. Multiple imputation techniques and sensitivity analyses were undertaken to investigate possible biases arising from sample attrition.

**Results:**

Grandparents looking after grandchildren, whether intensively or nonintensively, experienced some health benefits. Associations strengthened when attrition was accounted for, particularly if it is assumed that those who dropped out of the study were in poor health.

**Discussion:**

Our results show better health among grandparents who provided grandchild care in the European countries studied. These results are important given the widespread provision of grandchild care in Europe.

Social, economic, and demographic changes across Europe and the United States point to an increasing role for grandparents in providing childcare support to families (Gray, 2005). This is thought to be due to policies encouraging more mothers into the paid workforce, increases in divorce rates and single motherhood, and financial pressures on families (Aassve, Arpino, & Goisis, 2012; Herlofson & Hagestad, 2012). At the same time, promoting the health and well-being of older people is a critical policy imperative as populations age. Although studies generally show that grandparents provide vital support to families looking after grandchildren, the impacts of caring for grandchildren on the health of grandparents are inconclusive.

It is recognized that looking after grandchildren may be demanding, both physically and emotionally (Grinstead, Leder, Jensen, & Bond, 2003); however, provision of grandchild care may also be positively affirming and rewarding as grandparents may enjoy a closer relationship with their grandchildren (Pruchno & McKenney, 2002). Even after controlling for socioeconomic and demographic characteristics, and for previous health status, the effect of grandchild care on grandparent’s health seems to depend on its intensity, the cultural context, as well as on its stability and change. New and robust understandings of the ways grandchild care affects older adults will provide important evidence to enable policy makers across Europe to ensure that the role of grandparents in children’s lives is better supported and any deleterious effects on health are minimized.

Our aim is to examine the impact of caring for grandchildren on the health and well-being of grandparents in Europe using longitudinal data from the Survey of Health, Ageing and Retirement in Europe (SHARE), taking into account potential biases arising from attrition. Moreover, unlike many previous large U.S. studies that have focused either on grandparents who have “primary responsibility” for raising a grandchild or on “custodial households” (where a grandparent lives with a grandchild also acting as primary carer), we define grandchild care more broadly as whether, and how intensively, grandparents look after a grandchild without the parents being present.

## Background

Researchers have become increasingly interested in grandparents in the last decade as populations age and their roles in society, care, and work have become more visible to policy makers. Although research in this arena is bedeviled by definitional issues and data constraints, it is clear that grandparents play an important role in looking after their grandchildren. In the United States, one in four children younger than 5 years has been cared for by grandparents in the previous month (Laughlin, 2013). In a study of 11 European countries, 58% of grandmothers looked after at least one of their grandchildren aged 15 or younger in the preceding year in the absence of parents (Hank & Buber, 2009). Moreover, increasing coresidence between grandparents and grandchildren in the United States (from 3.2% of children in 1970 to 5.5% of children by 2003) suggests a rise in the share of grandparents raising or helping to raise grandchildren (U.S. Census Bureau, 2004).

Caring for grandchildren may have both positive and negative health effects. Role strain theory postulates that multiple roles are associated with poor health outcomes because of the psychological and physical stressors caused by demanding and potentially competing role responsibilities (Goode, 1960). For instance, if an individual’s obligations exceed his/her physical and psychological capacity to cope, this may cause an increase in stress and physical demands which in turn may be detrimental for health. This may be the case for those grandparents who act as primary carers or who provide full-time care for their grandchildren. Role enhancement theory suggests that those occupying multiple roles are more likely to be in better health than those with fewer responsibilities, as additional roles may provide individuals with a sense of usefulness and competence, enhancing control and reinforcing meaning in later life (Sieber, 1974). Engaging in a variety of roles may not only increase emotional exchanges and provide new opportunities for self-expression but also lead to physiological effects that help prevent chronic conditions (Holmes & Joseph, 2011). Grandparents who provide occasional grandchild care may therefore benefit from the emotional rewards and gratification stemming from this activity, which in turn may have a positive effect on health.

Research to date on the relationship between grandparent care and health and well-being is inconclusive. Early studies showed both a negative association between grandparental care and health problems, with poor physical and psychological health among grandparents with primary care responsibility for a grandchild (Grinstead et al., 2003; Minkler & Fuller-Thomson, 2001, 2005), and a positive relationship, with some grandparent caregivers describing higher quality of life and better health including weight loss and smoking cessation (Breeze & Stafford, 2010; Jendrek, 1993; Minkler, Roe, & Price, 1992). However, the health differences reported in these cross-sectional and often small-scale studies may reflect variations in socioeconomic status rather than in caregiving per se (Breeze & Stafford, 2010; Grinstead et al., 2003; Jendrek, 1993; Minkler & Fuller-Thomson, 2001, 2005; Minkler, Roe, & Price, 1992).

Fewer studies that have investigated the longitudinal relationship between grandparental childcare and health (largely based on U.S. data) have also led to mixed results. Several studies have found a relationship between grandparent childcare and depressive symptoms (Blustein, Chan, & Guanais, 2004; Minkler, Fuller-Thomson, Miller, & Driver, 1997) as well as physical health problems such as hypertension and coronary heart disease (Hayslip & Kaminski, 2005; Lee, Colditz, Berkman, & Kawachi, 2003), particularly among grandparents with primary care responsibilities or who coreside with grandchildren (Minkler & Fuller-Thomson, 2005; Minkler et al., 1997). Other studies have found beneficial effects or no major widespread health effects once previous characteristics (and prior health status in particular) are taken into account (Chen, Mair, Bao, & Yang, 2014; Hughes, Waite, LaPierre, & Luo, 2007).

Moreover, the relationship between grandparental childcare and health appears to be affected by the societal context. For example, in Taiwan and China, researchers did not find a negative effect of coresidence with grandchildren on grandparent health per se. Ku and colleagues (2013) found that coresident grandparents in Taiwan were more likely to report better self-rated health (SRH). Tsai, Motamed, and Rougemont (2013) also found that in Taiwan grandparents providing grandchild care were less likely to feel lonely and reported fewer depressive symptoms than those not providing any grandchild care. Chen and Liu (2012), using the longitudinal China Health and Nutrition Survey, found no differences in SRH between coresiding and noncoresiding grandparents; however, coresiding grandparents who provided more than 15hr per week of grandchild care were more likely to report worse SRH. A recent study also using SHARE data found that looking after a grandchild had a positive effect on the verbal fluency of grandparents in Europe, although no statistically significant effects were found for numeracy and recall (Arpino & Bordone, 2014)

Researchers have also examined the long-term effects of grandparental childcare by exploring the relationship between stability and change in grandparental childcare and health where, once again, the evidence is mixed. For example, several studies found that grandparents who took on grandchild care and those who increased their level of caregiving experienced greater negative health effects, including worsening physical and mental health, than those who did not transition to grandchild care (or to higher intensity care) (Baker & Silverstein, 2008; Hughes et al., 2007; Musil et al., 2011). However, some studies found that grandparents who recently started providing grandchild care or continued to provide nonintensive care reported better SRH, fewer functional limitations, and fewer depressive symptoms compared with grandparents who were not providing such care (Hughes et al., 2007; Ku et al., 2013).

Our study thus contributes to our knowledge in this area in several important ways. First, despite the widespread provision of childcare by grandparents in Europe, work on grandparenting and its health implications is sparse and most of the current studies are cross-sectional (Arpino & Bordone, 2014; Di Gessa, Glaser, Price, Ribe, & Tinker, 2015; Glaser, Di Gessa, & Tinker, 2014; Hank & Buber, 2009). Thus, we investigated the longitudinal associations between grandchild caregiving in Europe and three important indicators of health (SRH, depressive symptoms, and disability) both 2 and 4 years later, thereby allowing preexisting socioeconomic conditions and accounting for a wide variety of health indicators.

Second, although previous studies have tended to focus on custodial or primary grandchild carers, we were able to consider the more common supplementary grandparental childcare (i.e., complementary to parental care) taking into account its intensity as well as its stability and change over time, and controlling for living arrangements. The intensity level of grandparental childcare is important as prior studies have suggested that high levels of such care may have a negative effect on grandparents’ health (Chen & Liu, 2012; Hughes et al., 2007; Minkler & Fuller-Thomson, 2001), whereas lower levels are more likely to be positively associated with grandparents’ health (Tsai et al., 2013).

Finally, much previous longitudinal research is based on analyses of complete-record data sets only and does not consider how sample attrition might potentially bias associations (Fitzgerald, Gottschalk, & Moffit, 1998). In order to directly examine the effects of attrition in the sample studied, we used multiple imputation (MI) techniques.

Although the European countries in SHARE provide the context for this study, we nevertheless recognize that considerable variation exists within Europe with respect to grandparents’ characteristics and the level of childcare provided. For example, grandparents are significantly older in the Southern European countries (e.g., median age of 70 years in Greece) in comparison with the Nordic countries such as Denmark and Sweden (e.g., where the median age of grandparents is around 66 years; for more details on differences in grandparents’ characteristics by country, see Di Gessa et al. (2015) and Glaser et al. (2013)). Moreover, grandparental childcare varies considerably across Europe with higher levels of grandparental childcare found in those European countries (such as Italy and Spain) with lower levels of paid employment among older women, limited availability of formal childcare, and more conservative attitudes toward gendered family roles (Di Gessa et al., 2015; Igel & Szydlik, 2011; Jappens & Van Bavel, 2012). As health is likely to be affected by both variations in individual characteristics, such as age, and the interaction between individual and contextual factors (e.g., grandparents who regularly look after grandchildren in countries with few formal supports may be more likely to experience adverse health effects), it is important that the role of both these factors in the relationship between grandparental care and health is acknowledged.

## Method

### Study Population

We based our study on SHARE, a multidisciplinary longitudinal survey of individuals aged 50 and older. Details of the survey’s sampling frames and methodology, weighting strategies, and questionnaires have been reported elsewhere (http://www.share-project.org/). Data were drawn from the first four waves of the surveys. The first wave of SHARE took place in 2004/05 with later waves conducted biennially. In our study, we used data from Austria, Belgium, Switzerland, Germany, Denmark, Spain, France, Italy, the Netherlands, and Sweden, as these countries participated in all four waves. Analyses were restricted to respondents who had at least one grandchild at Wave 1, with an initial sample size of *N* = 15,374 grandparents. Study dropout in SHARE is high: 42% of respondents had dropped out of the survey by the second wave, and 51% had dropped out by the fourth wave. Our study restricted analyses to grandparents who were present in all waves but also considered possible bias arising from attrition.

### Measures

#### Outcome

Our key health outcomes were SRH, depressive symptoms, and disability. SRH and depressive symptoms were measured using well-validated scales (Idler & Benyamini, 1997; Prince et al., 1999). SRH was measured using a 5-point ordinal scale (*excellent*, *very good*, *good*, *fair*, or *poor*) across all four waves. We dichotomized the five SRH items into “fair or poor” versus better health, as previous studies have shown that morbidity and mortality are associated with adverse SRH (Idler & Benyamini, 1997). Depressive symptoms were measured using the EURO-D 12-item scale, whose validity and reliability has been demonstrated in cross-cultural context (Prince et al., 1999). Respondents were asked whether they had experienced any depressive symptoms, such as being unhappy or having trouble sleeping, recently or in the month prior to interview. We classified those who reported four or more depressive symptoms on the EURO-D scales as reporting depressive symptoms. The cutoff of a score of 4 or more as an indicator of depressive symptomatology has previously been validated against a variety of relevant clinical assessments in Europe (Prince et al., 1999). SHARE respondents were also asked a series of questions about whether they experienced any difficulties with basic activities of daily living (ADLs), such as bathing and eating, expected to last more than 3 months. We considered respondents who reported at least one difficulty to have an ADL disability (Katz, Ford, Moskowitz, Jackson, & Jaffe, 1963). Depressive symptoms and ADL disability, however, were not measured in Wave 3.

#### Measures of Grandchild Care

In Waves 1 and 2, respondents who had at least one grandchild were asked whether they looked after grandchildren without parents being present in the 12 months preceding the interview. If they did, they were also asked how often, on average, they looked after their grandchildren (i.e., almost daily, almost every week, almost every month, or less often) and then for how many hours (i.e., on a typical day/in a typical week/in a typical month/in the last 12 months depending on the response to the earlier question on frequency). Using this information, we distinguished two types of grandparental childcare: intensive (i.e., those who looked after at least one grandchild almost daily or for at least 15hr a week) and nonintensive care (i.e., those who looked after a grandchild weekly but for less than 15hr per week, monthly or less often). We chose this threshold for intensive grandparental childcare because these grandparents looked after their grandchildren on average for 30hr per week, roughly equivalent to holding a full-time job (Di Gessa et al., 2015; Fuller-Thomson & Minkler, 2001). Nonintensive provision of grandchild care groups together grandparents with similarly low levels of care (averaging less than about 1hr of care per working day).

We also created a 7-category measure of stability and change in the provision of grandchild care between baseline and Wave 2. We distinguished those who (i) continued to provide intensive or (ii) nonintensive care for grandchildren at both waves; (iii) were not providing care for a grandchild at either time point; (iv) took up the activity (no grandchild care provided at Wave 1 but intensive or nonintensive care provided at Wave 2); (v) ended their caregiving responsibilities between the two waves (providing any grandchild care at Wave 1 but not at Wave 2); (vi) provided less care (from intensive grandchild care at Wave 1 to nonintensive at Wave 2); and (vii) provided higher levels of care (from nonintensive at Wave 1 to intensive at Wave 2).

#### Other Covariates

Previous studies have shown SRH, depressive symptoms, and disability, as well as provision of grandparental childcare, to be associated with gender and age; socioeconomic and employment status, and educational level; participation in social activities; grandchild characteristics (e.g., number and age of grandchildren) and living arrangements; as well as other health measures and health behaviors, such as cognitive function, diabetes, obesity, and smoking (Glaser et al., 2010). In addition, given widely documented differences in health and grandparental childcare across Europe, we also included country fixed effects in our analyses (Crimmins, Kim, & Solé-Auró, 2011; Di Gessa et al., 2015; Hank & Buber, 2009).

We measured wealth using quintiles based on the harmonized sum of the net value of properties, nonhousing financial wealth, and business assets created by the RAND Corporation (for further details see www.mmicdata.rand.org/meta). We captured respondents’ employment status as being in paid work, retired or “other” (i.e., “unemployed,” “permanently sick or disabled,” “homemaker,” or “other”). We re-coded educational qualifications into three categories using the International Standard Classification of Education, where a low educational level is defined as being below a secondary education and high educational level refers to a university education or above (http://www.uis.unesco.org/). We defined participation in social activities as being involved in volunteering, training courses, political or religious organizations, or sport, social or other kind of clubs almost every week or more often. We considered both the age of the youngest grandchild and the total number of grandchildren as continuous variables. We measured living arrangements using a 4-category indicator, distinguishing between grandparents who lived alone, with at least one adult child, with their grandchildren (whether their parents were present), or in other types of living arrangements (i.e., mostly living with a spouse or partner only).

In addition to SRH, depressive symptoms, and ADL disability, we assessed health at baseline also using a variety of indicators, including cognitive index quintiles; chronic conditions (such as stroke and diabetes); obesity; and smoking. Cognitive ability was assessed by several questions relating to orientation in time (with questions about the interview date and the day of the week); verbal fluency (respondents were asked to name as many animals as they could think of in 1min); numeracy skills (with arithmetical calculations that assess how people use numbers in everyday life); and word recall (respondents were asked to recall aloud as many words as possible from a list of 10 words read by the interviewer). Combining the scores of all the tests, we created a binary indicator categorizing respondents as reporting poor cognitive function, if they scored in the lowest country-specific quintile for all tests (Singh-Manoux et al., 2010). In addition, we created binary indicators of doctor reported stroke or diabetes. We also measured obesity (BMI ≥ 30) and smoking (whether respondents were current smokers) as dichotomous variables.

### Statistical Analyses

We carried out preliminary analyses separately for men and women. We recognize that grandparenting is a gendered experience carrying different expectations for behaviors and responsibilities for men and women, which in turn may have different effects on health (Stelle, Fruhauf, Orel, & Landry-Meyer, 2010). However, in our multivariate model, it was not possible to run separate models for grandmothers and grandfathers because of the small numbers of grandfathers providing intensive childcare in each country (less than 30 grandfathers looked after their grandchildren intensively in Denmark, Sweden, the Netherlands, and Switzerland for example). In addition to the intensity of care, we also considered more detailed measures of provision and change in grandparental childcare (including distinguishing between respondents who moved from no grandchild care to nonintensive or intensive care, as well as those providing grandchild care to only one grandchild or more than one). However, the results of the multivariate analyses were broadly the same regardless of the measure of provision and change in grandchild care. Hence, we present results for the sample as a whole, and for the more simplified measures of provision of grandparental childcare, and its stability and change discussed earlier.

Our analyses of the impact of grandparental childcare consisted of two steps. First, we assessed the impact of grandparents’ childcare provision at Wave 2 on SRH at both Waves 3 and 4 and on depressive symptoms and ADL disability at Wave 4, controlling for baseline health, as well as for demographic and socioeconomic factors. Controlling for baseline characteristics, while focusing on childcare provision at Wave 2, permits us to some extent to take into account the associations between grandparents’ sociodemographic and health characteristics and the provision of grandchild care. Second, we investigated longitudinal associations between stability and change in grandchild care between baseline and Wave 2 and health at follow-up (i.e., SRH at Waves 3 and 4; depressive symptoms and ADL at Wave 4), controlling for the same baseline characteristics as described earlier.

We initially only controlled for each key health outcome at baseline; however, we also took into account other important health variables, introducing them into the model one by one in order to check for potential collinearity. The final model with all the health variables at baseline is presented here. Given the dichotomous nature of the outcome variables, we used logistic regression models. To take into account the complex sample designs, all of the analyses used the appropriate design weights provided by the SHARE teams.

We initially restricted the analyses described earlier to respondents with complete data on all the variables examined. As evidence suggests that patterns of attrition are likely to bias results (as in this case those who drop out of the study are more likely to be in the worst health), in a second stage, we used MI under the missing at random (MAR) assumption (i.e., we considered missingness to depend on fully observable variables in the data set; Little & Rubin, 2002) to explore the effects of missing data on the association between grandparental childcare and health. In this analysis, we imputed provision of grandchild care at Wave 2, SRH at Waves 3 and 4, and depressive symptoms and ADL disability at Wave 4 separately by country and gender using Multivariate Imputation by Chained Equations (MICE), including in the imputation all covariates considered in the analyses (such as age, education, wealth, and health). The chained equation process was continued for 20 cycles, and 200 imputed data sets were created. The results of analyses for each individual data set were then combined using Rubin’s rules (Little & Rubin, 2002). Given that MICE operates under the assumption that missing data are MAR, we also carried out sensitivity analyses in order to assess whether, and if so how, various plausible “arbitrary” assumptions about the missing data may affect the results. We tested the robustness of the results using pattern mixture models (Daniels & Hogan, 2008), that is by running successive analyses assuming that grandparents who dropped out of the study would have reported a 20% and 33% higher level of poor or fair SRH, depressive symptoms, or ADL disability than their counterparts who remained in the study. This is because other studies have shown that those in poor health are indeed more likely to drop out of longitudinal studies (Behr, Bellgardt, & Rendtel, 2005; Fitzgerald et al., 1998). All analyses were performed using Stata 13.

## Results

### Descriptive Statistics

Table 1 shows the health of older grandparents and their socioeconomic and demographic characteristics at baseline by provision of grandparental childcare. Overall, just more than half of grandparents (52%) looked after their grandchildren in the preceding year with 12% providing intensive grandchild care (i.e., almost daily or at least 15hr a week) and close to 40% providing grandchild care on a nonintensive basis. Table 1 also shows that grandparents who were not looking after grandchildren were more likely to report worse health, whereas those who provided nonintensive levels of grandchild care generally reported better health. Overall, more highly educated younger grandparents, those in paid work, and who were socially engaged, were more likely to provide nonintensive childcare.

**Table 1.** Distribution of Grandparent Baseline Characteristics, by Type of Grandchild Care at Wave 2

Variables at baseline	Grandchild care at Wave 2			
	Total SHARE	No care	Nonintensive care	Intensive care
Outcome variables				
SRH fair/poor	29.0	35.2	21.4	29.1
With depressive symptoms	24.6	28.1	19.5	27.6
ADL disability	9.5	12.6	6.4	6.8
Control variables				
Female (%)	58.4	56.7	58.4	64.6
Age (mean)	66.6	70.6	63.0	62.4
Education (middle)	27.6	24.9	31.1	26.7
Education (high)	18.0	13.5	24.0	15.8
In paid work	19.2	10.9	28.7	20.9
Retired	58.5	67.9	49.6	50.5
Other	22.3	21.1	21.6	28.6
In lowest quintile of wealth	17.9	21.5	13.8	17.4
Engaged in social activities	28.2	23.4	34.9	23.3
Age of youngest grandchild (mean)	8.4	11.9	5.2	5.0
Number of grandchildren (mean)	4.3	4.5	4.1	3.9
Living with adult child(ren)	13.3	12.1	13.6	17.2
Living alone	19.1	28.4	15.4	11.4
Living with grandchild	2.5	2.0	1.1	7.2
Living with spouse/partner or others	64.8	57.5	70.0	64.2
Diabetes	10.2	12.3	7.8	9.9
In lowest cognitive function	15.2	23.1	7.8	8.2
Stroke	4.0	5.3	3.2	1.9
Obesity (BMI ≥ 30)	18.7	18.7	17.5	22.7
Smoker	15.5	13.6	17.7	16.1
Number of observations (*N*)	8,485	4,078	3,369	1,038
%	100.0	48.1	39.7	12.2

*Notes:* ADL = activities of daily living; SRH = self-rated health. Sources: SHARE, 2004 and 2006. Own calculation.

Table 2 shows stability and change in grandchild care between baseline and Wave 2. The majority of grandparents (65%) provided some type of grandchild care between waves. Around one third of grandparents at Wave 2 continued to provide the same level of childcare reported at baseline: 27% continued to provide nonintensive and 7% continued to provide intensive childcare. Around 14% of grandparents increased their level of childcare between waves (i.e., either providing no care at baseline and any care at Wave 2 or providing nonintensive childcare at baseline and intensive childcare at Wave 2), and a similar percentage ended their caregiving responsibilities (with 4% reducing their level of childcare between the waves).

**Table 2.** Distribution of Stability and Change in Grandchild Care Between Waves 1 and 2

Stability and change in grandparental childcare	%
Continued nonintensive childcare at both waves	26.7
No childcare at either wave	35.3
No childcare at baseline → Any childcare at Wave 2	10.6
Continued intensive childcare	6.7
Stopped childcare	12.8
Nonintensive childcare → Intensive childcare	3.6
Intensive → Nonintensive childcare	4.4
Number of observations at Wave 2 (*N*)	8,485

*Note:* Sources: SHARE, 2004 and 2006. Own calculation.

### Associations Between Caregiving and Health Indicators at Follow-Ups

Table 3 shows results from logistic regression models that investigated associations between provision of grandchild care at Wave 2 and SRH, depressive symptoms, and ADL disability at 2- and 4-year follow-up (i.e., at Waves 3 and 4), controlling for baseline socioeconomic and demographic characteristics and health. Table 3 is based on respondents with complete data only across all waves.

**Table 3.** Association Between Grandparental Childcare at Wave 2 and Fair or Poor SRH, Depressive Symptoms, and ADL Disability at 2- and 4-Year Follow-Up, Controlling for Baseline Health, Socioeconomic, and Demographic Characteristics

	SRH W3		SRH W4		Depressive symptoms W4		ADL disability W4	
	OR	95% CIs	OR	95% CIs	OR	95% CIs	OR	95% CIs
Female	0.98	0.88–1.09	0.96	0.83–1.09	1.78**	1.54–2.05	0.85	0.70–1.03
60–69^a^	0.95	0.77–1.16	1.30**	1.07–1.59	1.00	0.80–1.25	1.72**	1.25–2.35
70–79^a^	1.20	0.91–1.58	1.72**	1.37–2.16	1.17	0.92–1.49	2.95**	2.07–4.19
80+^a^	1.14	0.78–1.66	1.97**	1.43–2.71	1.50*	1.08–2.09	6.71**	4.41–10.2
Mid education^b^	0.96	0.81–1.13	0.96	0.84–1.12	0.87	0.75–1.02	1.16	0.87–1.53
High education^b^	0.83**	0.70–0.99	0.78*	0.63–0.95	0.79*	0.66–0.95	1.06	0.78–1.42
In paid work^c^	0.57*	0.45–0.72	0.68**	0.52–2.90	0.87	0.68–1.12	0.94	0.60–1.47
Other work^c^	1.06	0.88–1.27	1.18	0.98–1.42	1.10	0.92–1.30	1.28*	1.02–1.61
II wealth quintile^d^	0.90	0.76–1.08	0.90	0.73–1.11	0.80*	0.66–0.98	0.86	0.63–1.17
III wealth quintile^d^	0.75*	0.63–0.90	0.90	0.73–1.10	0.82	0.67–1.01	0.83	0.63–1.07
IV wealth quintile^d^	0.71*	0.59–0.86	0.69**	0.57–0.84	0.85	0.68–1.07	0.76*	0.58–0.99
Highest wealth quintile^d^	0.65*	0.52–0.81	0.63**	0.49–0.81	0.80	0.64–1.01	0.76	0.54–1.06
Engaged in social activities	0.73**	0.64–0.84	0.83**	0.73–0.95	0.86	0.73–1.02	0.81*	0.65–0.99
Number of grandchildren	1.01	0.99–1.03	1.02	0.99–1.04	1.02	0.99–1.04	1.04**	1.01–1.06
Age of youngest grandchild	1.01	0.99–1.02	1.00	0.99–1.01	1.01	0.99–1.02	1.01	0.99–1.02
With adult children^e^	0.81	0.66–1.00	0.92	0.75–1.14	1.04	0.84–1.30	1.19	0.91–1.56
Alone^e^	1.02	0.85–1.23	0.99	0.85–1.16	0.84*	0.72–0.98	1.34**	1.10–1.62
Living with grandchild^e^	1.54	0.97–2.48	1.21	0.76–1.93	0.97	0.55–1.69	1.26	0.69–2.31
Nonintensive childcare^f^	0.85	0.71–1.02	0.86*	0.75–0.97	0.94	0.78–1.13	0.86	0.69–1.07
Intensive childcare^f^	0.77*	0.62–0.96	0.89	0.70–1.13	0.90	0.72–1.12	0.87	0.65–1.16
SRH fair/poor^g^	5.25**	4.32–6.37	3.99**	3.44–4.63	1.73**	1.48–2.01	1.99**	1.62–2.43
1 + ADL disability	1.80**	1.45–2.23	1.59**	1.21–2.08	1.17	0.90–1.53	3.81**	3.00–4.83
With depressive symptoms^h^	1.71**	1.47–2.00	1.80**	1.55–2.10	4.08**	3.54–4.71	1.51**	1.26–1.81
Lowest cognitive function	1.06	0.88–1.27	1.30**	1.06–1.60	1.21	0.95–1.53	1.63**	1.29–2.05
Diabetes	1.88**	1.45–2.44	1.79**	1.44–2.23	1.14	0.92–1.41	1.56**	1.22–2.00
Stroke	1.83**	1.34–2.50	2.08**	1.40–3.09	1.26	0.87–1.83	2.14**	1.47–3.10
Obese	1.54**	1.30–1.82	1.39**	1.17–1.63	0.97	0.83–1.14	1.83**	1.49–2.23
Smoker	1.45**	1.21–1.72	1.39**	1.19–1.64	1.30**	1.09–1.57	1.27	0.96–1.67
Austria	1.10	0.75–1.59	0.79	0.56–1.13	0.48**	0.30–0.74	1.21	0.76–1.92
Germany	1.28	0.95–1.72	1.22	0.92–1.62	0.77	0.54–1.09	1.32	0.87–2.01
Sweden	1.18	0.92–1.50	0.85*	0.67–0.99	0.64**	0.48–0.87	1.31	0.95–1.89
Netherlands	0.68*	0.50–0.92	0.71*	0.52–0.98	0.45**	0.32–0.64	0.62	0.37–1.02
Spain	1.25	0.91–1.72	1.16	0.85–1.57	0.88	0.62–1.26	1.18	0.79–1.80
Italy	1.15	0.87–1.51	1.16	0.87–1.55	1.00	0.71–1.41	1.15	0.74–1.79
Denmark	0.75**	0.63–0.89	0.53**	0.44–0.64	0.47**	0.37–0.59	0.85	0.65–1.11
Switzerland	0.63**	0.51–0.76	0.57**	0.46–0.71	0.48**	0.37–0.62	1.43	0.99–1.96
Belgium	0.70**	0.55–0.88	0.66**	0.51–0.85	0.77	0.57–1.03	1.42*	1.00–2.02
Constant	0.48**	0.64–0.84	0.34**	0.24–0.49	0.03**	0.02–0.05	0.21**	0.14–0.33
Number of observations	6,224		5,381		5,333		5,380	

*Notes:* ADL = activities of daily living; CI = confidence interval; OR = odds ratio; SRH = self-rated health. ORs and 95% CIs obtained from fully adjusted logistic regression. Sources: SHARE Waves 1, 2, 3, and 4.

Reference categories: ^a^50–59; ^b^Lowest educational group; ^c^Retired; ^d^Lowest wealth quintile; ^e^Living with spouse/partner or others; ^f^No grandchild care provided; ^g^SRH = good, very good, or excellent; ^h^No or fewer than four depressive symptoms reported on the EURO-D scale. All models also included country fixed effects controls (with France as reference).

*Significant at the .05 level. **Significant at the .0l level. Own calculation.

When SRH is considered, both at Waves 3 and 4, Table 3 shows significant differences between grandparents who provided some type of grandchild care and those who did not provide any childcare at Wave 2. For instance, provision of intensive grandchild care was significantly associated with lower odds of reporting fair or poor SRH 2 years later, whereas nonintensive grandchild care was significantly associated with lower odds of reporting fair or poor SRH after 4 years. No significant associations between the provision of any grandchild care and subsequent depressive symptomatology and ADL disability were found. Associations with other baseline covariates were broadly similar as would be expected from previous studies. Grandmothers were more likely to report depressive symptoms than grandfathers. Older grandparents were significantly more likely to report poor health, depressive symptoms, and ADL disability; grandparents with higher educational levels were significantly less likely to report fair or poor SRH and depressive symptoms compared with those with the lowest education level. Being in the highest wealth quintiles was significantly associated with a lower likelihood of reporting fair or poor SRH. There was also a reverse association between paid work, engagement in social activities, and fair or poor SRH; similarly, grandparents engaged in social activities were less likely to report ADL disability at follow-up. In all models, as expected, baseline health and health behaviors were strongly associated with fair or poor SRH and ADL disability at follow-up. Moreover, grandparents’ household composition was not significantly associated with subsequent health (with the exception of living alone at baseline, which was significantly associated with higher ADL disability but lower levels of depressive symptoms at follow-up). Finally, even when this broad set of grandparents’ socioeconomic, demographic, and health-related characteristics is controlled for in the multivariate analysis, substantial differences in country coefficients are found: grandparents in Denmark, Switzerland, Belgium, the Netherlands, and Sweden were less likely to report fair or poor SRH and depressive symptoms at follow-up compared with French grandparents. No significant differences were found across the other countries.

Table 4 shows the associations involving changes in grandparental childcare between baseline and Wave 2 and subsequent health once baseline socioeconomic, demographic, and health characteristics were taken into account. For all three outcomes, stability or change in grandparental childcare was not associated with subsequent health; however, there was some evidence to suggest that grandparents who did not provide any grandchild care at either wave were more likely to report fair or poor SRH at Wave 4 compared with those who continued to look after their grandchildren nonintensively between baseline and Wave 2.

**Table 4.** Association Between Stability and Change in Grandparental Childcare Between Baseline and Wave 2 and Poor SRH, Depressive Symptoms, and ADL Disability at 2- and 4-Year Follow-Up, Controlling for Baseline Health, Socioeconomic, and Demographic Characteristics

	SRH W3		SRH W4		Depressive symptoms W4		ADL disability W4	
	OR	95% CIs	OR	95% CIs	OR	95% CIs	OR	95% CIs
Nonintensive childcare at both waves	Ref		Ref		Ref		Ref	
No childcare at either wave	1.22	0.96–1.55	1.21*	1.00–1.47	1.25	0.93–1.67	1.10	0.90–1.34
No childcare at baseline → Any childcare at Wave 2	1.04	0.69–1.28	1.04	0.87–1.24	0.99	0.65–1.51	1.02	0.79–1.31
Continued intensive childcare	0.85	0.65–1.12	0.96	0.73–1.27	0.94	0.64–1.39	1.01	0.76–1.33
Stopped childcare	1.09	0.89–1.35	1.13	0.92–1.39	1.02	0.75–1.40	1.09	0.83–1.43
Nonintensive childcare → Intensive childcare	0.91	0.66–1.26	1.09	0.76–1.59	1.09	0.65–1.81	0.82	0.55–1.21
Intensive → Nonintensive childcare	0.94	0.72–1.23	1.26	0.91–1.75	1.09	0.65–1.80	1.32	0.95–1.83
Number of observations	6,212		5,368		5,367		5,320	

*Notes:* ADL = activities of daily living; CI = confidence interval; OR = odds ratio; SRH = self-rated health. ORs and 95% CIs obtained from fully adjusted logistic regression. Sources: SHARE Waves 1, 2, 3, and 4. Controlling for age, gender, education, wealth, employment status, social engagement, number of grandchildren, age of youngest grandchild, living arrangements, country, SRH, depressive symptoms, ADL disability, lowest cognitive function quintile, diabetes, stroke, obesity, and smoking at baseline.

*Significant at the .05 level. **Significant at the .0l level. Own calculation.

### Imputation and Sensitivity Analysis

The results reported earlier come from complete-record analyses. As sample attrition was considerable, we repeated regression analyses for each outcome under the assumption that nonresponders at Waves 3 and 4 were MAR. We also performed sensitivity analyses under the assumption that those who dropped out of the study reported 20% and 33% higher levels of poor health than respondents, regardless of baseline characteristics. Table 5 shows that under MAR, grandparents who provided any grandchild care were less likely to report adverse health outcomes (broadly similar to results in Table 3). However, the worse the health attributed to those who dropped out of the study, the stronger and more significant the associations between grandparental childcare and better health at follow-up become. In particular, under both scenarios of missing not at random (MNAR), associations between intensive grandparental childcare at Wave 2 and depressive symptoms and ADL disability at Wave 4 strengthened and became significant at the 5% level. Provision of nonintensive grandchild care was significantly associated with lower odds of reporting depressive symptoms and ADL disability only under the 33% scenario. As for living arrangements, results under MAR show that grandparents living with an adult child, those living with their grandchildren, and those who lived alone at baseline were all more likely to report ADL disability 6 years later compared with those living with their spouse only; however, these associations weakened under the assumption of MNAR.

**Table 5.** Association Between Grandparental Childcare at Wave 2 and Fair or Poor SRH, Depressive Symptoms, and ADL Disability at 2- and 4-Year Follow-Up, Controlling for Baseline Health, Socioeconomic, and Demographic Characteristics

	SRH W3						SRH W4					
	MAR		MNAR 20%		MNAR 33%		MAR		MNAR 20%		MNAR 33%	
	OR	95% CIs	OR	95% CIs	OR	95% CIs	OR	95% CIs	OR	95% CIs	OR	95% CIs
With adult children^a^	0.93	0.80–1.08	0.91	0.78–1.05	0.89	0.77–1.03	0.97	0.79–1.17	0.95	0.81–1.11	0.93	0.80–1.08
Alone^a^	0.96	0.84–1.09	0.94	0.82–1.08	0.93	0.80–1.08	0.91	0.79–1.05	0.94	0.81–1.10	0.93	0.79–1.09
Living with grandchild^a^	1.19	0.86–1.66	1.20	0.86–1.67	1.22	0.87–1.69	1.13	0.76–1.68	1.08	0.77–1.52	1.11	0.80–1.54
Nonintensive childcare^b^	0.86**	0.76–0.98	0.86**	0.75–0.97	0.85***	0.76–0.96	0.86**	0.76–0.98	0.87**	0.76–0.98	0.86**	0.76–0.97
Intensive childcare^b^	0.83**	0.69–1.00	0.83**	0.69–0.98	0.83**	0.69–0.98	0.86	0.72–1.04	0.85	0.71–1.03	0.84*	0.70–1.02
	Depressive symptoms W4						ADL W4					
	MAR		MNAR 20%		MNAR 33%		MAR		MNAR 20%		MNAR 33%	
	OR	95% CIs	OR	95% CIs	OR	95% CIs	OR	95% CIs	OR	95% CIs	OR	95% CIs
With adult children^a^	1.08	0.90–1.28	1.05	0.89–1.24	1.03	0.88–1.21	1.35***	1.09–1.66	1.18	0.95–1.47	1.12	0.92–1.35
Alone^a^	0.89	0.76–1.04	0.88	0.75–1.04	0.87	0.74–1.03	1.33***	1.12–1.59	1.18	0.93–1.50	1.11	0.89–1.40
Living with grandchild^a^	0.91	0.62–1.32	0.95	0.66–1.37	0.97	0.68–1.38	1.75***	1.16–2.64	1.51**	1.01–2.24	1.43	0.99–2.07
Nonintensive childcare^b^	0.87	0.74–1.02	0.87*	0.76–1.01	0.87**	0.76–1.00	0.83	0.67–1.03	0.85*	0.72–1.01	0.86**	0.74–1.00
Intensive childcare^b^	0.82	0.66–1.03	0.82**	0.67–0.99	0.81**	0.67–0.98	0.76	0.57–1.02	0.78**	0.61–0.97	0.79**	0.63–0.97

*Notes:* ADL = activities of daily living; MAR = missing at random; MNAR = missing not at random; SRH = self-rated health. Odds ratios and 95% confidence intervals obtained from fully adjusted logistic regression with imputed data sets under MAR and MNAR (assuming SRH as fair or poor, depressive symptoms and ADL disability among nonrespondents increased by 20% and 33% respectively). Sources: SHARE Waves 1, 2, 3, and 4.

Reference categories: ^a^Living with spouse/partner or others; ^b^No grandchild care provided. Controlling for age, gender, education, wealth, work status, social engagement, number of grandchildren, age of youngest grandchild, country, SRH, ADL disability, lowest cognitive function, depressive symptoms, diabetes, stroke, obesity, and smoking at baseline.

*Significant at the .10 level. **Significant at the .05 level. ***Significant at the .0l level. Own calculation.

When considering associations between changes and stability of childcare provision between baseline and Wave 2 and the health outcomes at follow-ups, both under MAR and MNAR we found that for all three health outcomes only grandparents who did not provide any grandchild care at both waves were consistently and significantly more likely to report poorer SRH, depressive symptoms, and ADL disability at follow-up than grandparents who provided nonintensive grandchild care at both waves (results not shown). This is similar to the results shown in Table 4 that considered respondents with complete data only.

## Discussion

As grandparents play an increasingly significant role in family life, particularly looking after grandchildren, it is important to assess whether grandparental childcare affects grandparents’ health. Our aim was to assess the impact of childcare provision on SRH, depressive symptoms, and ADL disability among older grandparents in Europe. As previous research has shown variations in health effects by the intensity of grandchild care provided, as well as in changes in grandparental childcare over time, we examined these associations distinguishing between intensive (i.e., daily childcare or for at least 15hr per week) and less intensive grandparental childcare. Our longitudinal results, based on respondents with complete data on all indicators considered here, showed positive associations between grandchild care and better SRH: grandparents who provided intensive grandchild care were less likely to report poor or fair health after 2 years compared with those who did not provide any care, whereas those who cared for their grandchildren nonintensively were less likely to report fair or poor health 4 years later. No significant associations were found between grandparental childcare and depressive symptoms or ADL disability at follow-up. Although findings from complete data analyses are likely to be affected by attrition (as discussed later), our results from these analyses provide evidence that grandparents looking after grandchildren, whether intensively or nonintensively, experience some health benefits, even when prior health, socioeconomic, and demographic characteristics are taken into account. Further, our results (once again based on those respondents with no missing information on any of the indicators considered here) also showed no differences between grandparents who increased, decreased, or continued grandchild care provision between baseline and Wave 2: we found limited evidence that grandparents who did not look after grandchildren at both waves were more likely to report poor or fair health.

Our analyses focused on grandparents in Europe, and controlled for country-level effects given that the provision of intensive or nonintensive grandparental childcare is likely to be affected by contextual–structural and cultural factors other than individual demographic, health, and socioeconomic characteristics. Results suggest disparities in health between countries, with Danish, Dutch, and Swedish grandparents generally reporting better self-perceived health. However, such health patterns have been observed in previous studies and are also likely to reflect broader differences in the magnitude and generosity of varying welfare state regimes (such as policies aimed at reducing social inequalities and relative poverty) as well as cultural differences in the way older people report their health (Crimmins, Kim, & Solé-Auró, 2011; Eikemo, Bambra, Judge, & Ringdal, 2008).

SHARE is affected by high attrition rates as more than half of grandparents (51%) had dropped out by the fourth wave. Attrition cannot be ignored as it is likely to bias results, particularly when the outcome of interest—health—is likely to be a key factor associated with grandparents who both drop out of the study and take on caregiving roles. That is, grandparents in poor health are thought to be more likely to drop out of the survey and they are also less likely to look after grandchildren, leaving a sample of grandparent caregivers in better health in the study. MI techniques and sensitivity analyses confirmed that understanding sample attrition is informative and not considering its impact may lead to underestimating observed associations. For example, if we assume that those grandparents who dropped out of the study were 33% more likely to report poor or fair health, both intensive and nonintensive grandparental childcare at Wave 2 became significantly associated (at the 5% level) with SRH at Wave 3. Similarly, under MNAR, grandparents providing intensive and nonintensive grandchild care become significantly less likely to report poor or fair SRH, to have depressive symptoms, and to have ADL limitations after 4 years.

Overall, our results suggest that looking after grandchildren has a positive association with grandparents’ health over time, even when previous health is taken into account. However, the causal relationship between provision of grandchild care and “good health” is difficult to identify, even in longitudinal studies. Although in this study we control for baseline health and socioeconomic characteristics to attempt to account for initial selection in looking after grandchildren, it is plausible that better health is a trigger for providing grandchild care and that this advantage is maintained over time. Moreover, several mechanisms may explain the positive relationship between provision of childcare and health. Looking after grandchildren may provide grandparents with emotional gratification and a sense of usefulness and competence, thereby enhancing life satisfaction and strengthening their role fulfillment (Sieber, 1974). Given that our study considers the more common supplementary grandchild care (i.e., complementary to parental care), it is likely that grandparents’ involvement in such a family activity may provide them with a sense of belonging, value, and attachment, thereby enhancing intergenerational relationships and social ties with both grandchildren and their parents. Consistent with previous studies on family support and relationships and their effect on health, it is plausible that grandparents providing childcare may benefit from greater emotional, instrumental, and social support from their adult children (Hayslip, Blumenthal, & Garner, 2014; House, Landis, & Umberson, 1988). Family networks and support may also have a direct positive impact on health by promoting healthy behaviors and providing occasions for positive emotional exchanges which may act to buffer the potential negative effects of caregiving. Finally, looking after grandchildren may help to increase or maintain physical activity among grandparents, which is associated with improved well-being and physical health as well as a reduction in the symptoms of anxiety and depression (Goodwin, 2003; Holmes & Joseph, 2011).

### Strengths and Limitations

We investigated longitudinal associations between provision of grandchild care and health using large-scale nationally representative European data. Given that almost 40% of grandparents in SHARE provided nonintensive childcare, and about 12% looked after their grandchildren intensively, our finding that provision of grandchild care is not associated with poor health is noteworthy. Our study’s contributions include a broader definition of grandchild care that considers the more common supplementary grandparental childcare rather than the more limited custodial or primary grandchild care. Furthermore, in our study we accounted for both living arrangements and the intensity of grandchild care. In addition, we also explicitly paid attention to missingness and to possible biases arising from attrition, an unavoidable problem in longitudinal studies.

Our analyses, however, have some limitations. The measurements considered rely on self-reports: measurements such as SRH, as well as the number of hours of care provided, may be sensitive to cultural norms and differences in definitions. Moreover, our measure of nonintensive grandchild care is limited as it includes grandparents who looked after grandchildren for less than 15hr a week, monthly or less often making the interpretation of this indicator more difficult. Furthermore, the SHARE questionnaire did not provide us with information that would have enabled us to capture the full complexity of grandparental childcare: we know nothing about the quality of childcare provided, whether grandparents gained satisfaction from it, and the meanings that they may attach to such activity. Also, we were not able to control for the quality of the relationship between grandparents and the parents of the grandchildren they looked after, and we had no information on what grandparents actually do when they provide care. Similarly, we do not know how involved grandparents were in the decision to look after grandchildren. Grandparents were not asked whether they chose to or had to look after grandchildren: if grandparents looked after grandchildren because they wanted to, it is likely that such an activity would be perceived as being more rewarding and beneficial for health. Also, it may be that grandparents with more resources, more time, and better health are more likely to be approached by parents to provide grandchild care.

Related to the lack of data on the particular experiences of grandparent caregivers, this study did not explicitly study gender differences. Grandfathers and grandmothers perform care differently and may have different expectations of involvement (Stelle et al., 2010). Although we acknowledge that variations in grandchild care by gender may have a different effect on the health of grandmothers and grandfathers, data constrains hindered us from running separate models to explore whether similar longitudinal associations were found for both sexes.

In addition, although in our analyses we controlled for the age of the youngest grandchild, the latter may not necessarily be the one that grandparents were looking after. It is known that, even in the same household, the relationship grandparents have with their grandchildren, the activities they share with them, and their style of interactions are likely to depend on which grandchild they look after and on their age (Mueller & Elder, 2003). For instance, the activities grandparents do when they provide care (such as working on projects together, providing the grandchild with an opportunity to learn the grandparent’s skills, attending plays, or playing in the park) are likely to depend on the age of the grandchild they actually look after and have different effects on grandparents’ health.

Finally, although in our analyses we took country-level differences into account, we did not investigate cross-national differences in the relationship between provision of grandchild care and grandparents’ health. However, we acknowledge that the relationship between grandparental childcare and health may vary across countries. For instance, grandparents who provide childcare in countries where such support is not expected, and where formal childcare provision is widespread, may attach different meanings to such activity in comparison with their counterparts in societies where family care is preferred and little formal support is available. Such variations in the context in which grandchild care occurs are thus likely to lead to different effects on health.

Further work is needed to identify causal pathways underlying the association between grandparental childcare and health, taking into account the circumstances surrounding the onset of caregiving. However, if looking after grandchildren is beneficial for grandparents’ health, more attention should be paid to those factors associated with childcare provision, as younger, healthier, and financially better-off grandparents are more likely to take care of their grandchildren particularly in the absence of conflicting commitments such as paid work (Di Gessa et al., 2015).

## Funding

This research was supported by the UK Economic and Social Research Council (ESRC) grant ES/K003348/1, in partnership with Grandparents Plus, the Calouste Gulbenkian Foundation, and the Beth Johnson Foundation.
